# A Retrospective Study on the Antimicrobial Susceptibility Patterns of Elizabethkingia meningoseptica Specimens Among Adult Patients

**DOI:** 10.7759/cureus.99220

**Published:** 2025-12-14

**Authors:** Ruchita Sahu, Basanti Kumari Pathi, Kumudini Panigrahi, Smrutisree Mohapatra, Jyoti Prakash Sahoo

**Affiliations:** 1 Microbiology, Kalinga Institute of Medical Sciences, Bhubaneswar, IND; 2 Pharmacology, Kalinga Institute of Medical Sciences, Bhubaneswar, IND

**Keywords:** antibiogram pattern, antimicrobial susceptibility pattern, chryseobacterium, elizabethkingia, extended-spectrum beta-lactamase (esbl), healthcare-associated infection, hospital-acquired infection, malditof, metallo beta-lactamase, vitek 2

## Abstract

Background and objectives: *Elizabethkingia meningoseptica* has evolved as one of the emerging and multidrug-resistant organisms globally. We planned this study to evaluate the antimicrobial susceptibility testing (AST) patterns of *E*.* meningoseptica* isolates in our hospital. We also gauged the AST patterns of the isolates from various samples and patients with short (i.e., ≤ 28 days) and prolonged (i.e., > 28 days) hospitalization.

Methods: This retrospective study was conducted from August 2023 to July 2025 at Kalinga Institute of Medical Sciences (KIMS), Bhubaneswar, India. We analyzed the data of all adult inpatients with positive culture reports for *E*.* meningoseptica*. All specimens (blood, endotracheal tube (ETT), urine, sputum, wound, pus, body fluid, and skin swabs) were analyzed to evaluate the AST findings of the concerned pathogen. The subgroup analyses were done as per the sample (i.e., blood, ETT, and others) and length of hospitalization (≤ 28 and > 28 days). We illustrated the collected data via mosaic and chord diagrams. The VITEK 2 system was employed for evaluating AST. We used R software (version 4.5.2) for data analysis.

Results: We assessed 208 samples that were positive for *E*.* meningoseptica* isolates. The median age of the study population was 62.0 (46.0-71.0) years. Male patients were predominant in our study (133, 63.94%). Blood (109, 52.40%) and ETT (63, 30.29%) were the most frequently positive samples for *E. meningoseptica*. Thirty-six (17.31%) of the positive samples came from urine, skin swabs, sputum, nasal swabs, wounds, and pus. The average length of stay in the hospital was 22.0 (14.0-33.0) days. A total of 72 participants (34.62%) endured more than 28 days in the hospital. Of the 208 individuals, 65 (31.25%) died, and 143 (68.75%) were discharged. The isolates demonstrated the highest sensitivity towards minocycline (148, 71.2%), followed by levofloxacin (87, 41.8%) and imipenem (84, 40.4%). No sample exhibited colistin sensitivity. Over 80% of the samples showed resistance to most antimicrobials. For ceftriaxone and aztreonam, the resistance was 100%. Drug resistance to minocycline was the lowest (48, 23.1%). The subgroup analyses were similar.

Conclusion: Blood and ETT samples accounted for the majority of *E*.* meningoseptica* isolates. The isolates were highly sensitive to minocycline, levofloxacin, and imipenem. The isolates were more resistant to aztreonam, ceftriaxone, piperacillin-tazobactam, and amikacin, in contrast to other antibiotics.

## Introduction

*Elizabethkingia meningoseptica* (*E*. *meningoseptica*), also referred to as *Flavobacterium meningosepticum* and *Chryseobacterium meningosepticum*, is an aerobic, gram-negative, non-fermentative, nonmotile, catalase-, and oxidase-positive bacterium [1-4]. The first human case of *E*. *meningoseptica* infection was documented in 1959 by Elizabeth O. King [2,3,5]. It is an opportunistic pathogen that mostly infects individuals with impaired immune systems, sepsis, diabetes, hypertension, and cancers [6-8]. Nowadays, *E*. *meningoseptica* infections are progressively rising to the status of potentially fatal nosocomial infections globally [8-11].

*Elizabethkingia* species are widely distributed in water reservoirs and soil [12]. *E*. *meningoseptica* frequently colonizes sink basins and taps, which could serve as reservoirs for nosocomial infections [12,13]. Compared to other species in the same genus, *E*. *meningoseptica* is more virulent. The infections caused by it are challenging to treat and can be fatal [1,13,14]. The two types of β-lactamases found in *E*. *meningoseptica* are class A extended-spectrum β-lactamases (ESBLs) and class B metallo-β-lactamases (MBLs) [15,16]. MBLs cause resistance to carbapenems, whereas ESBLs impart resistance to cephalosporins [17]. *E*. *meningoseptica* infection has increased the mortality rate up to 65.6% and 66.7% in adult and pediatric patients, respectively [1,12,18].

We have limited treatment options for *E*. *meningoseptica* infection, as it is intrinsically resistant to multiple categories of antibiotics, including colistin [3,19,20]. A plausible cause for the emergence of this resistance pathogen is the overuse of colistin and other broad-spectrum antimicrobials [3,21]. Additionally, the exceptional capacity of *E*. *meningoseptica* to develop resistance against antimicrobials and disinfectants allows it to spread hospital-acquired infections (HAIs) [2-4,21]. Our previous studies have discovered the multidrug resistance of *E*. *meningoseptica*, despite its very low incidence among patients admitted to neurosurgery units [22,23]. Therefore, we conducted a retrospective study to gauge the epidemiology of infections caused by *E*. *meningoseptica* among adult patients admitted to our hospital. We analyzed the AST data of *E*. *meningoseptica* isolates detected in the study population, including those with short (i.e., ≤ 28 days) and prolonged (i.e., > 28 days) hospital stays, as well as positive samples (i.e., blood, ETT, and others).

## Materials and methods

This retrospective study was conducted to analyze the AST patterns of *E*. *meningoseptica* in adult patients admitted to KIMS between August 2023 and July 2025. Before the study commenced, we obtained ethical clearance from the Institutional Ethics Committee (KIIT/KIMS/IEC/2285/2025, dated August 18, 2025). The study adhered to the Strengthening the Reporting of Observational Studies in Epidemiology (STROBE) guidelines, institutional norms, Good Laboratory Practices, and the Declaration of Helsinki.

Study criteria

We screened the laboratory data of adult patients admitted during the above-mentioned time period and had culture reports positive for *E*. *meningoseptica*. The cultures obtained from pediatric patients and outpatients were excluded.

Study procedure

Blood, ETT, and other samples (e.g., urine, skin swabs, sputum, nasal swabs, wounds, and pus) were collected and sent to the microbiological laboratory. Only a single sample was collected from each of the participants. Body fluid and respiratory samples were inoculated into MacConkey agar and blood agar after Gram staining. They were then placed in an incubator overnight with 5% CO_2_. The BacT/Alert 3D device (BioMérieux, Marcy-l'Étoile, France) was used to incubate blood samples and other fluids. Following a positive flag, they were grown on sheep blood agar and MacConkey agar. Cystine-lactose-electrolyte-deficient agar was used to inoculate urine samples. Pus, wound, nasal, and skin swabs were inoculated using blood agar and MacConkey agar. The VITEK 2 compact system (BioMérieux, Marcy-l'Étoile, France) GN card 406 was used to identify and perform an AST on non-lactose-fermenting transparent colonies of Elizabethkingia that demonstrated positive oxidase and non-fermentative O-F-test results. The AST results were examined using the 2024 cut-off values established by the Clinical and Laboratory Standards Institute (CLSI) [24].

Statistical analysis

Convenience sampling was adopted in this retrospective study. The normality of the data distribution was assessed using the Kolmogorov-Smirnov test. The median and interquartile range (IQR) were determined for the continuous data. Similarly, frequency and proportion were calculated for the categorical data. To illustrate the distribution of participants, we constructed a mosaic plot. The mosaic plot was segmented by specimen type (blood, ETT, or others), gender (male or female), age group (adult or elderly), and outcome (discharge or death). Chord diagrams were used to display the AST patterns of *E*. *meningoseptica* isolates from the entire study population and various subgroups. For data analysis and plot creation, R (R Foundation for Statistical Computing, Vienna, Austria) version 4.5.2 [25] was utilized.

## Results

In this retrospective study, we analyzed only the 208 culture reports (positive for *E*. *meningoseptica*) from adult patients admitted to KIMS between August 2023 and July 2025. The demographic details of the participants are displayed in Table 1. The study population’s median age was 62.0 (46.0-71.0) years. Eighty-one (38.94%) participants were above 65 years of age. There was a male preponderance (133, 63.94%) in our study. The most common sample positive for *E*. *meningoseptica* was blood (109, 52.40%), followed by ETT (63, 30.29%). Other samples, such as urine, skin swabs, sputum, nasal swabs, wounds, and pus, accounted for 36 (17.31%) positive samples. The median duration of hospitalization was 22.0 (14.0-33.0) days. Seventy-two (34.62%) participants were hospitalized for more than 28 days. Of the 208 participants, 143 (68.75%) were discharged, and 65 (31.25%) succumbed to death.

**Table 1:** Demographic characteristics of the study participants The categorical and continuous variables are displayed as frequency with percentage and median with IQR, respectively. IQR: interquartile range, ETT: endotracheal tube.

Parameter	Value
Total participants	208
Age (years)	62.0 (46.0-71.0)
Age > 65 years	81 (38.94%)
Male	133 (63.94%)
Duration of hospitalization (days)	22.0 (14.0-33.0)
Hospital stay > 28 days	72 (34.62%)
Samples collected (positive for Elizabethkingia meningoseptica)	
Blood	109 (52.40%)
ETT	63 (30.29%)
Others	36 (17.31%)
Outcome	
Discharge	143 (68.75%)
Death	65 (31.25%)

**Figure 1:**
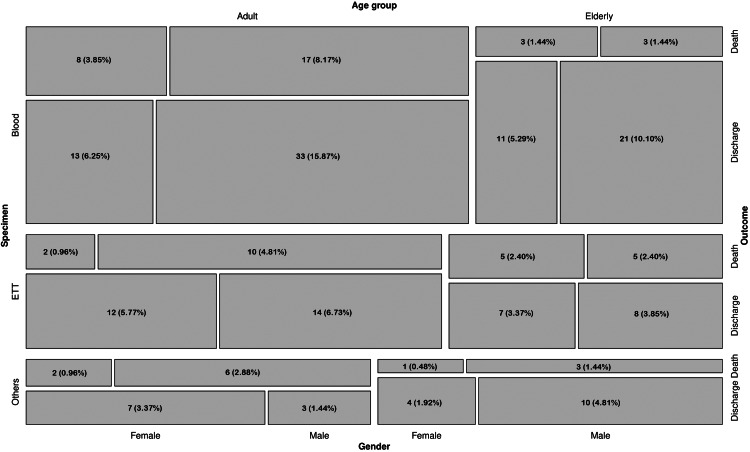
Distribution of the study participants The mosaic plot illustrates the distribution of the study populations based on specimen (blood, ETT, or others), age group (adult or elderly), outcome (discharge or death), and gender (male or female). specimen (shown on the left Y-axis), followed by age group (shown on the top X-axis), outcome (shown on the right Y-axis), and gender (shown on the bottom X-axis). ETT: endotracheal tube.

Figure 1 illustrates the distribution of the study population through a mosaic plot. We divided the study population based on the specimen (blood, ETT, or others), age group (adult or elderly), outcome (discharge or death), and gender (male or female). The order of the segregation was specimen (shown on the left Y-axis), followed by age group (shown on the top X-axis), outcome (shown on the right Y-axis), and gender (shown on the bottom X-axis). The numbers and percentages of participants in each block are mentioned in the mosaic plot. The largest block represents the adult male patients with blood samples positive for *E*. *meningoseptica* who got discharged (33, 15.87%), followed by the elderly male patients with blood samples positive for *E*. *meningoseptica* who got discharged (21, 10.10%), the adult male patients with blood samples positive for *E*. *meningoseptica* who died during hospitalization (17, 8.17%), the adult male patients with ETT samples positive for *E*. *meningoseptica* who got discharged (14, 6.73%), and the adult female patients with blood samples positive for *E*. *meningoseptica* who got discharged (13, 6.25%).

**Figure 2:**
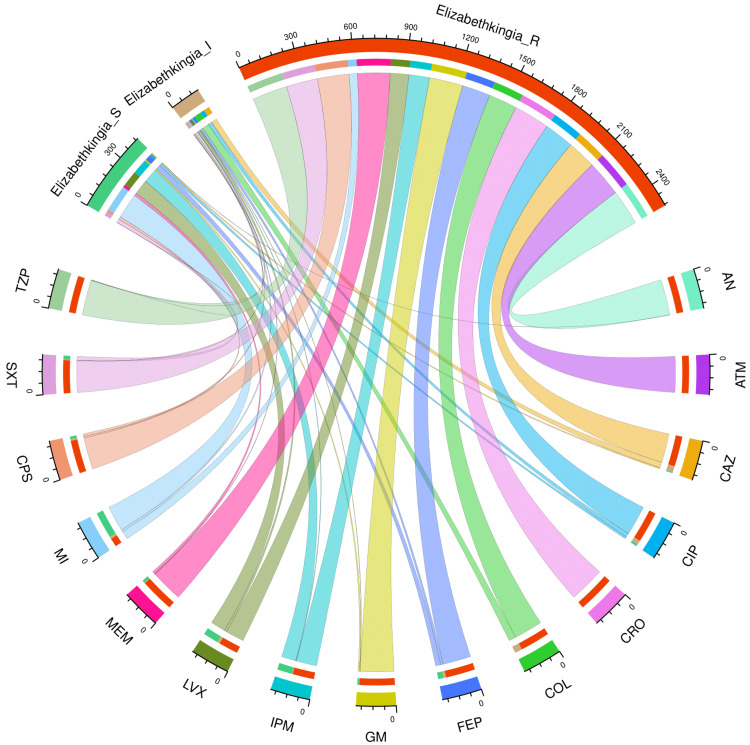
AST findings of Elizabethkingia meningoseptica isolates found among the study population (n = 208) The lower and upper portions denote 15 antimicrobials (shown in different colours) and three types of antimicrobial susceptibility (S: sensitive, I: intermediate, and R: resistant) of *Elizabethkingia meningoseptica* specimens detected in the study population (n = 208). The band widths represent the number of *Elizabethkingia meningoseptica* specimens and their AST profiles for the 15 drugs. The curved scales running along the arcs represent the total number of cases for the corresponding arcs. Here, the total number of cases is 208 (specimens) x 15 (antimicrobials) = 3120. AST: antimicrobial susceptibility testing, AN: amikacin, ATM: aztreonam, CAZ: ceftazidime, CIP: ciprofloxacin, CRO: ceftriaxone, COL: colistin, FEP: cefepime, GM: gentamicin, IPM: imipenem, LVX: levofloxacin, MEM: meropenem, MI: minocycline, CPS: cefoperazone-sulbactam, SXT: cotrimoxazole, TZP: piperacillin-tazobactam.

Figure 2 and Table 2 portray the AST profile of *E*. *meningoseptica* specimens detected in the study participants (n = 208). The chord diagram in Figure 2 illustrates the sensitivity of the 208 *E*. *meningoseptica* isolates found in the study population to 15 antimicrobials, categorized in the upper and lower sections, respectively. The curved scales running along the arcs represent the total number of cases for the corresponding arcs. Here, the total number of cases is 208 (specimens) x 15 (antimicrobials) = 3120. The highest sensitivity was seen towards minocycline (148, 71.2%), followed by levofloxacin (87, 41.8%) and imipenem (84, 40.4%). No sample showed sensitivity towards colistin. More than 80% of the specimens demonstrated resistance to the majority of antimicrobials. The resistance was 100% for aztreonam and ceftriaxone. The drug resistance was the least against minocycline (48, 23.1%).

**Table 2:** AST findings of Elizabethkingia meningoseptica isolates found among the study population (n = 208) The AST findings are presented as numbers and percentages. AST: antimicrobial susceptibility testing, AN: amikacin, ATM: aztreonam, CAZ: ceftazidime, CIP: ciprofloxacin, CRO: ceftriaxone, COL: colistin, FEP: cefepime, GM: gentamicin, IPM: imipenem, LVX: levofloxacin, MEM: meropenem, MI: minocycline, CPS: cefoperazone-sulbactam, SXT: cotrimoxazole, TZP: piperacillin-tazobactam.

Drugs	Sensitive	Intermediate	Resistant
AN	1 (0.5%)	0	207 (99.5%)
ATM	0	0	208 (100%)
CAZ	7 (3.4%)	33 (15.9%)	168 (80.8%)
CIP	13 (6.3%)	22 (10.6%)	173 (83.2%)
CRO	0	0	208 (100%)
COL	0	41 (19.7%)	167 (80.3 %)
FEP	30 (14.4%)	14 (6.7%)	164 (78.8%)
GM	9 (4.3%)	3 (1.4%)	196 (94.2%)
IPM	84 (40.4%)	4 (1.9%)	120 (57.7%)
LVX	87 (41.8%)	13 (6.3%)	108 (51.9%)
MPM	16 (7.7%)	3 (1.4%)	189 (90.9%)
MI	148 (71.2%)	12 (5.8%)	48 (23.1%)
CPS	15 (7.2%)	9 (4.3%)	184 (88.5%)
SXT	22 (10.6%)	3 (1.4%)	183 (88%)
TZP	1 (0.5%)	3 (1.4%)	204 (98.1%)

Subgroup analysis based on the duration of hospitalization

**Figure 3:**
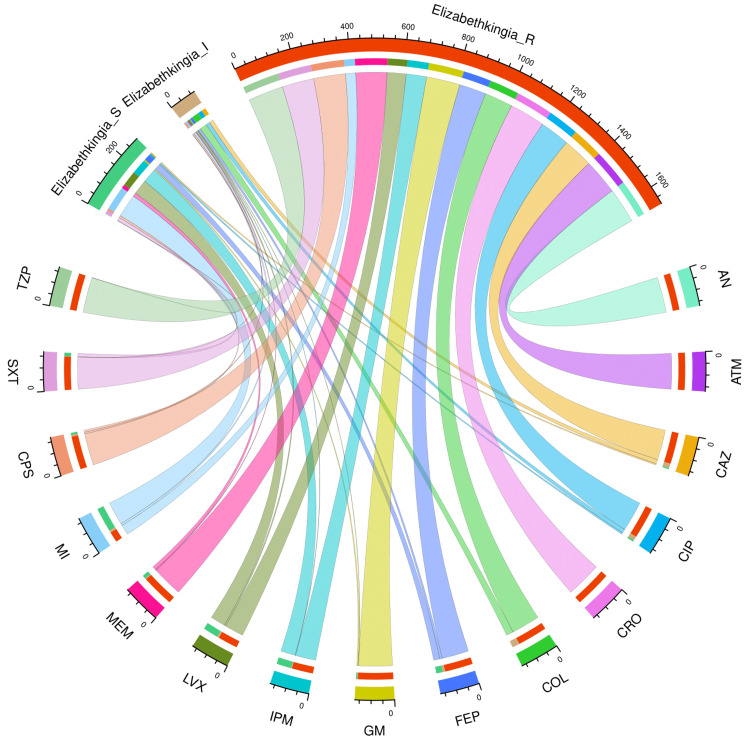
AST findings of Elizabethkingia meningoseptica isolates found among the participants with hospital stay ≤ 28 days (n = 136) The lower and upper portions denote 15 antimicrobials (shown in different colours) and three types of antimicrobial susceptibility (S: sensitive, I: intermediate, and R: resistant) of *Elizabethkingia meningoseptica* specimens detected in participants with a duration of hospitalization ≤ 28 days (n = 136). The band widths represent the number of *Elizabethkingia meningoseptica* specimens and their AST profiles for the 15 drugs. The curved scales running along the arcs represent the total number of cases for the corresponding arcs. Here, the total number of cases is 136 (specimens) x 15 (antimicrobials) = 2040. AST: antimicrobial susceptibility testing, AN: amikacin, ATM: aztreonam, CAZ: ceftazidime, CIP: ciprofloxacin, CRO: ceftriaxone, COL: colistin, FEP: cefepime, GM: gentamicin, IPM: imipenem, LVX: levofloxacin, MEM: meropenem, MI: minocycline, CPS: cefoperazone-sulbactam, SXT: cotrimoxazole, TZP: piperacillin-tazobactam.

Figure 3 and Table 3 present the AST profile of *E*. *meningoseptica* specimens detected in study participants with hospital stay duration ≤ 28 days (n = 136). The chord diagram in Figure 3 illustrates the sensitivity of the 136 *E*. *meningoseptica* isolates found among the participants with shorter hospital stays and 15 antimicrobials in the upper and lower sections, respectively. The curved scales running along the arcs represent the total number of cases for the corresponding arcs. Here, the total number of cases is 136 (specimens) x 15 (antimicrobials) = 2040. The highest sensitivity was seen towards minocycline (89, 65.4%), followed by levofloxacin (57, 41.9%), and imipenem (56, 41.2%). No sample showed sensitivity towards colistin. More than 80% of the specimens demonstrated resistance to the majority of antimicrobials. The resistance was 100% for aztreonam and ceftriaxone. The drug resistance was the least against minocycline (39, 28.7%).

**Table 3:** AST findings of Elizabethkingia meningoseptica isolates found among the study participants with hospital stay ≤ 28 days (n = 136) The AST findings are presented as numbers and percentages. AST: antimicrobial susceptibility testing, AN: amikacin, ATM: aztreonam, CAZ: ceftazidime, CIP: ciprofloxacin, CRO: ceftriaxone, COL: colistin, FEP: cefepime, GM: gentamicin, IPM: imipenem, LVX: levofloxacin, MEM: meropenem, MI: minocycline, CPS: cefoperazone-sulbactam, SXT: cotrimoxazole, TZP: piperacillin-tazobactam.

Drugs	Sensitive	Intermediate	Resistant
AN	0	0	136 (100%)
ATM	0	0	136 (100%)
CAZ	5 (3.7%)	16 (11.8%)	115 (84.6%)
CIP	6 (4.4%)	15 (11.0%)	115 (84.6%)
CRO	0	0	136 (100%)
COL	0	27 (19.9%)	109 (80.1%)
FEP	24 (17.6%)	9 (6.6%)	103 (75.7%)
GM	5 (3.7%)	2 (1.5%)	129 (94.9%)
IPM	56 (41.2%)	2 (1.5%)	78 (57.4%)
LVX	57 (41.9%)	6 (4.4%)	73 (53.7%)
MPM	14 (10.3%)	3 (2.2%)	119 (87.5%)
MI	89 (65.4%)	8 (5.9%)	39 (28.7%)
CPS	8 (5.9%)	6 (4.4%)	122 (89.7%)
SXT	13 (9.6%)	1 (0.7%)	122 (89.7%)
TZP	1 (0.7%)	0	135 (99.3%)

**Figure 4:**
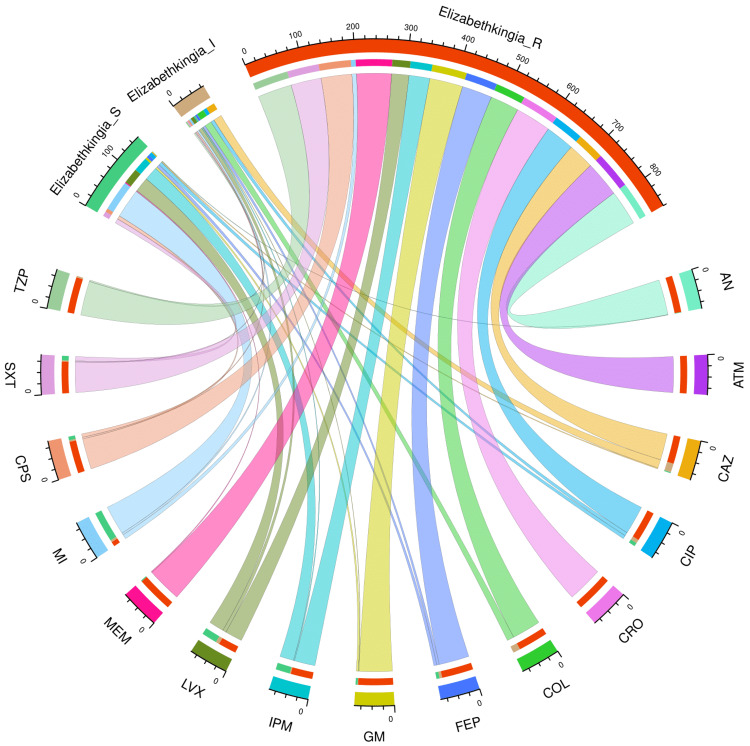
AST findings of Elizabethkingia meningoseptica isolates found among the participants with hospital stay > 28 days (n = 72) The lower and upper portions denote 15 antimicrobials (shown in different colours) and three types of antimicrobial susceptibility (S: sensitive, I: intermediate, and R: resistant) of *Elizabethkingia meningoseptica* specimens detected in participants with a duration of hospitalization greater than 28 days (n = 72). The band widths represent the number of *Elizabethkingia meningoseptica* specimens and their AST profiles for the 15 drugs. The curved scales running along the arcs represent the total number of cases for the corresponding arcs. Here, the total number of cases is 72 (specimens) x 15 (antimicrobials) = 1080. AST: antimicrobial susceptibility testing, AN: amikacin, ATM: aztreonam, CAZ: ceftazidime, CIP: ciprofloxacin, CRO: ceftriaxone, COL: colistin, FEP: cefepime, GM: gentamicin, IPM: imipenem, LVX: levofloxacin, MEM: meropenem, MI: minocycline, CPS: cefoperazone-sulbactam, SXT: cotrimoxazole, TZP: piperacillin-tazobactam.

Figure 4 and Table 4 present the AST profile of *E*. *meningoseptica* specimens detected in study participants with a hospital stay of more than 28 days (n = 72). The chord diagram in Figure 4 illustrates the sensitivity of the 72 *E*. *meningoseptica* isolates found among the participants with longer hospital stays and 15 antimicrobials in the upper and lower sections, respectively. The curved scales running along the arcs represent the total number of cases for the corresponding arcs. Here, the total number of cases is 72 (specimens) x 15 (antimicrobials) = 1080. The highest sensitivity was seen towards minocycline (59, 81.9%), followed by levofloxacin (30, 41.7%) and imipenem (28, 38.9%). No sample showed sensitivity towards colistin. More than 80% of the specimens demonstrated resistance to the majority of antimicrobials. The resistance was 100% for aztreonam and ceftriaxone. The drug resistance was the least against minocycline (9, 12.5%).

**Table 4:** AST findings of Elizabethkingia meningoseptica isolates found among the study participants with hospital stay > 28 days (n = 72) The AST findings are presented as numbers and percentages. AST: antimicrobial susceptibility testing, AN: amikacin, ATM: aztreonam, CAZ: ceftazidime, CIP: ciprofloxacin, CRO: ceftriaxone, COL: colistin, FEP: cefepime, GM: gentamicin, IPM: imipenem, LVX: levofloxacin, MEM: meropenem, MI: minocycline, CPS: cefoperazone-sulbactam, SXT: cotrimoxazole, TZP: piperacillin-tazobactam.

Drugs	Sensitive	Intermediate	Resistant
AN	1 (1.4%)	0	71 (98.6%)
ATM	0	0	72 (100%)
CAZ	2 (2.8%)	17 (23.6%)	53 (73.6%)
CIP	7 (9.7%)	7 (9.7%)	58 (80.6%)
CRO	0	0	72 (100%)
COL	0	14 (19.4%)	58 (80.6%)
FEP	6 (8.3%)	5 (6.9%)	61 (84.7%)
GM	4 (5.6%)	1 (1.4%)	67 (93.1%)
IPM	28 (38.9%)	2 (2.8%)	42 (58.3%)
LVX	30 (41.7%)	7 (9.7%)	35 (48.6%)
MPM	2 (2.8%)	0	70 (97.2%)
MI	59 (81.9%)	4 (5.6%)	9 (12.5%)
CPS	7 (9.7%)	3 (4.2%)	62 (86.1%)
SXT	9 (12.5%)	2 (2.8%)	61 (84.7%)
TZP	0	3 (4.2%)	69 (95.8%)

Subgroup analysis based on the samples

**Figure 5:**
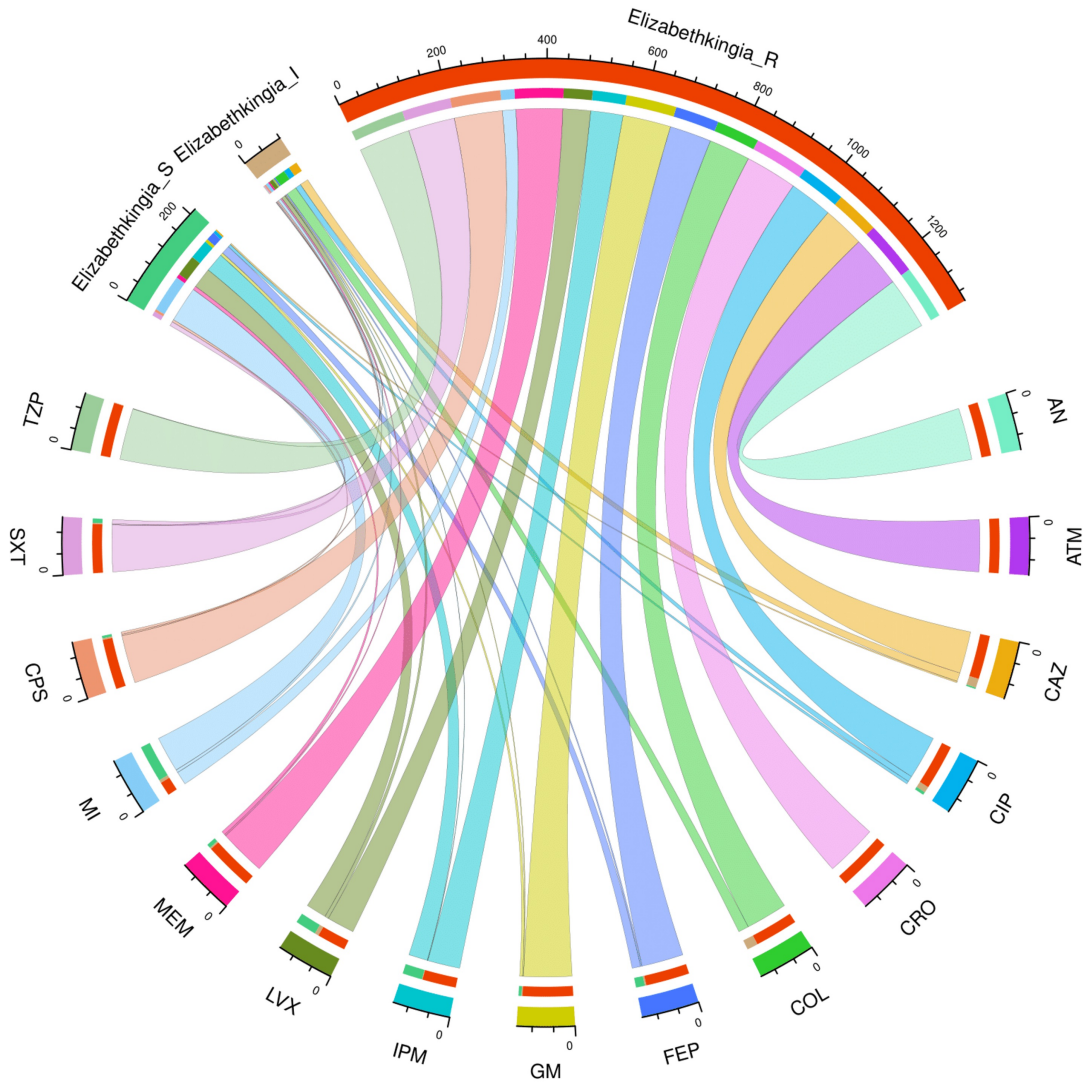
AST findings of Elizabethkingia meningoseptica isolates found in the blood samples of the study participants (n = 109) The lower and upper portions denote 15 antimicrobials (shown in different colours) and three types of antimicrobial susceptibility (S: sensitive, I: intermediate, and R: resistant) of *Elizabethkingia meningoseptica* specimens detected in the blood samples of the participants (n = 109). The band widths represent the number of *Elizabethkingia meningoseptica* specimens and their AST profiles for the 15 drugs. The curved scales running along the arcs represent the total number of cases for the corresponding arcs. Here, the total number of cases is 109 (specimens) x 15 (antimicrobials) = 1635. AST: antimicrobial susceptibility testing, AN: amikacin, ATM: aztreonam, CAZ: ceftazidime, CIP: ciprofloxacin, CRO: ceftriaxone, COL: colistin, FEP: cefepime, GM: gentamicin, IPM: imipenem, LVX: levofloxacin, MEM: meropenem, MI: minocycline, CPS: cefoperazone-sulbactam, SXT: cotrimoxazole, TZP: piperacillin-tazobactam.

Figure 5 and Table 5 portray the AST profile of *E*. *meningoseptica* isolates detected in the blood samples (n = 109). The chord diagram in Figure 5 illustrates the sensitivity of 109 *E*. *meningoseptica* isolates found in the blood samples of the participants to 15 antimicrobials, categorized in the upper and lower sections, respectively. The curved scales running along the arcs represent the total number of cases for the corresponding arcs. Here, the total number of cases is 109 (specimens) x 15 (antimicrobials) = 1635. The highest sensitivity was seen towards minocycline (75, 68.8%), followed by levofloxacin (43, 39.4%) and imipenem (40, 36.7%). No sample showed sensitivity towards colistin. More than 80% of the specimens demonstrated resistance to the majority of antimicrobials. The resistance was 100% for aztreonam and ceftriaxone. The drug resistance was the least against minocycline (28, 25.7%).

**Table 5:** AST findings of Elizabethkingia meningoseptica isolates found in the blood samples of the study participants (n = 109) The AST findings are presented as numbers and percentages. AST: antimicrobial susceptibility testing, AN: amikacin, ATM: aztreonam, CAZ: ceftazidime, CIP: ciprofloxacin, CRO: ceftriaxone, COL: colistin, FEP: cefepime, GM: gentamicin, IPM: imipenem, LVX: levofloxacin, MEM: meropenem, MI: minocycline, CPS: cefoperazone-sulbactam, SXT: cotrimoxazole, TZP: piperacillin-tazobactam.

Drugs	Sensitive	Intermediate	Resistant
AN	0	0	109 (100%)
ATM	0	0	109 (100%)
CAZ	3 (2.8%)	18 (16.5%)	88 (80.7%)
CIP	6 (5.5%)	12 (11.0%)	91 (83.5%)
CRO	0	0	109 (100%)
COL	0	22 (20.2%)	87 (79.8%)
FEP	18 (16.5%)	3 (2.8%)	88 (80.7%)
GM	6 (5.5%)	2 (1.8%)	101 (92.7%)
IPM	40 (36.7%)	1 (0.9%)	68 (62.4%)
LVX	43 (39.4%)	8 (7.3%)	58 (53.2%)
MPM	8 (7.3%)	3 (2.8%)	98 (89.9%)
MI	75 (68.8%)	6 (5.5%)	28 (25.7%)
CPS	5 (4.6%)	3 (2.8%)	101 (92.7%)
SXT	9 (8.3%)	2 (1.8%)	98 (89.9%)
TZP	0	1 (0.9%)	108 (99.1%)

**Figure 6:**
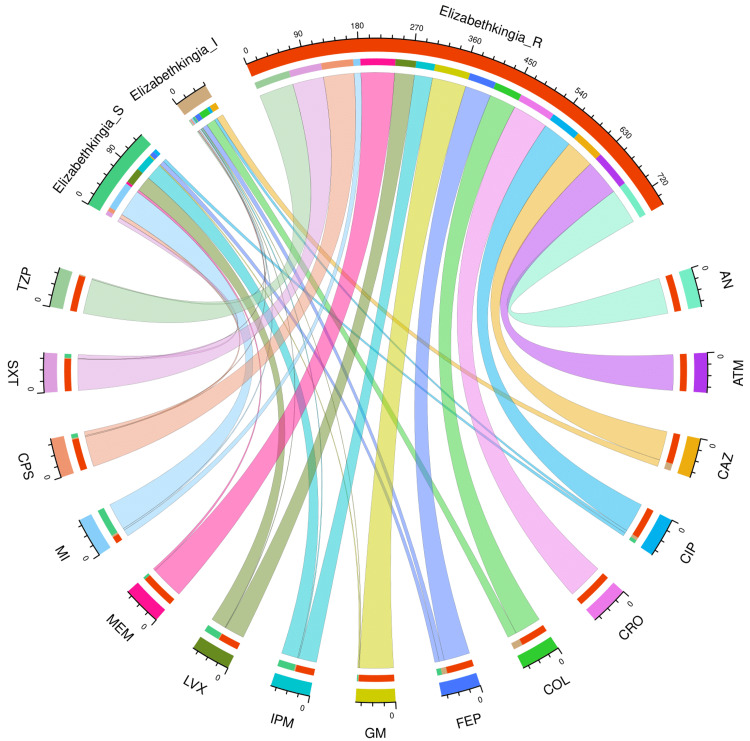
AST findings of Elizabethkingia meningoseptica isolates found in the ETT samples of the study participants (n = 63) The lower and upper portions denote 15 antimicrobials (shown in different colours) and three types of antimicrobial susceptibility (S: sensitive, I: intermediate, and R: resistant) of *Elizabethkingia meningoseptica* specimens detected in the ETT samples of the participants (n = 109). The band widths represent the number of *Elizabethkingia meningoseptica* specimens and their AST profiles for the 15 drugs. The curved scales running along the arcs represent the total number of cases for the corresponding arcs. Here, the total number of cases is 63 (specimens) x 15 (antimicrobials) = 945. ETT: endotracheal tube, AST: antimicrobial susceptibility testing, AN: amikacin, ATM: aztreonam, CAZ: ceftazidime, CIP: ciprofloxacin, CRO: ceftriaxone, COL: colistin, FEP: cefepime, GM: gentamicin, IPM: imipenem, LVX: levofloxacin, MEM: meropenem, MI: minocycline, CPS: cefoperazone-sulbactam, SXT: cotrimoxazole, TZP: piperacillin-tazobactam.

Figure 6 and Table 6 portray the AST profile of *E*. *meningoseptica* isolates detected in the ETT samples (n = 63). The chord diagram in Figure 6 illustrates the sensitivity of the 63 *E*. *meningoseptica* isolates found in the ETT samples of the participants to 15 antimicrobials, categorized in the upper and lower sections, respectively. The curved scales running along the arcs represent the total number of cases for the corresponding arcs. Here, the total number of cases is 63 (specimens) x 15 (antimicrobials) = 945. The highest sensitivity was seen towards minocycline (48, 76.2%), followed by imipenem (29, 46.0%), and levofloxacin (27, 42.9%). No sample showed sensitivity towards colistin. More than 80% of the specimens demonstrated resistance to the majority of antimicrobials. The resistance was 100% for aztreonam and ceftriaxone. The drug resistance was the least against minocycline (12, 19.0%).

**Table 6:** AST findings of Elizabethkingia meningoseptica isolates found in the ETT samples of the study participants (n = 63) The AST findings are presented as numbers and percentages. AST: antimicrobial susceptibility testing, AN: amikacin, ATM: aztreonam, CAZ: ceftazidime, CIP: ciprofloxacin, CRO: ceftriaxone, COL: colistin, FEP: cefepime, GM: gentamicin, IPM: imipenem, LVX: levofloxacin, MEM: meropenem, MI: minocycline, CPS: cefoperazone-sulbactam, SXT: cotrimoxazole, TZP: piperacillin-tazobactam.

Drugs	Sensitive	Intermediate	Resistant
AN	0	0	63 (100%)
ATM	0	0	63 (100%)
CAZ	0	12 (19.0%)	51 (81.0%)
CIP	6 (9.5%)	5 (7.9%)	52 (82.5%)
CRO	0	0	63 (100%)
COL	0	16 (25.4%)	47 (74.6%)
FEP	8 (12.7%)	9 (14.3%)	46 (73.0%)
GM	2 (3.2%)	1 (1.6%)	60 (95.2%)
IPM	29 (46.0%)	2 (3.2%)	32 (50.8%)
LVX	27 (42.9%)	1 (1.6%)	35 (55.6%)
MPM	4 (6.3%)	0	59 (93.7%)
MI	48 (76.2%)	3 (4.8%)	12 (19.0%)
CPS	7 (11.1%)	2 (3.2%)	54 (85.7%)
SXT	7 (11.1%)	1 (1.6%)	55 (87.3%)
TZP	0	2 (3.2%)	61 (96.8%)

**Figure 7:**
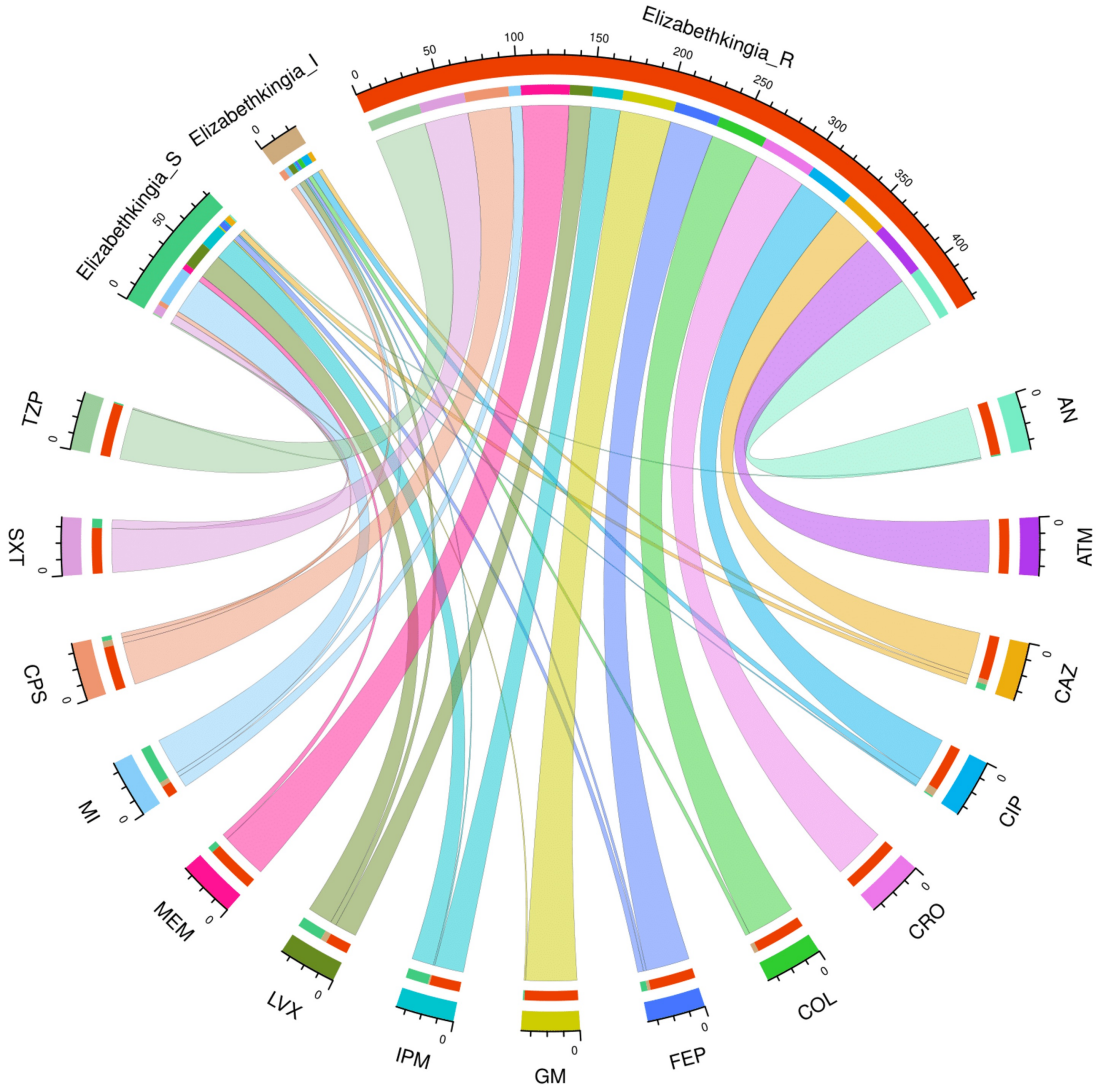
AST findings of Elizabethkingia meningoseptica isolates found in other samples of the study participants (n = 36) The lower and upper portions denote 15 antimicrobials (shown in different colours) and three types of antimicrobial susceptibility (S: sensitive, I: intermediate, and R: resistant) of *Elizabethkingia meningoseptica* specimens detected in other samples of the participants (n = 36). The band widths represent the number of *Elizabethkingia meningoseptica* specimens and their AST profiles for the 15 drugs. The curved scales running along the arcs represent the total number of cases for the corresponding arcs. Here, the total number of cases is 36 (specimens) x 15 (antimicrobials) = 540. AST: antimicrobial susceptibility testing, AN: amikacin, ATM: aztreonam, CAZ: ceftazidime, CIP: ciprofloxacin, CRO: ceftriaxone, COL: colistin, FEP: cefepime, GM: gentamicin, IPM: imipenem, LVX: levofloxacin, MEM: meropenem, MI: minocycline, CPS: cefoperazone-sulbactam, SXT: cotrimoxazole, TZP: piperacillin-tazobactam.

Figure 7 and Table 7 portray the AST profile of *E*. *meningoseptica* isolates detected in other samples (n = 36). The chord diagram in Figure 7 illustrates the sensitivity of the 36 *E*. *meningoseptica* isolates found in other samples of the participants and 15 antimicrobials in the upper and lower sections, respectively. The curved scales running along the arcs represent the total number of cases for the corresponding arcs. Here, the total number of cases is 36 (specimens) x 15 (antimicrobials) = 540. The highest sensitivity was seen towards minocycline (25, 69.4%), followed by levofloxacin (17, 47.2%), and imipenem (15, 41.7%). No sample showed sensitivity towards colistin. More than 80% of the specimens demonstrated resistance to the majority of antimicrobials. The resistance was 100% for aztreonam and ceftriaxone. The drug resistance was the least against minocycline (8, 22.2%).

**Table 7:** AST findings of Elizabethkingia meningoseptica isolates found in the other samples of the study participants (n = 36) The AST findings are presented as numbers and percentages. AST: antimicrobial susceptibility testing, AN: amikacin, ATM: aztreonam, CAZ: ceftazidime, CIP: ciprofloxacin, CRO: ceftriaxone, COL: colistin, FEP: cefepime, GM: gentamicin, IPM: imipenem, LVX: levofloxacin, MEM: meropenem, MI: minocycline, CPS: cefoperazone-sulbactam, SXT: cotrimoxazole, TZP: piperacillin-tazobactam.

Drugs	Sensitive	Intermediate	Resistant
AN	1 (2.8%)	0	35 (97.2%)
ATM	0	0	36 (100%)
CAZ	4 (11.1%)	3 (8.3%)	29 (80.6%)
CIP	1 (2.8%)	5 (13.9%)	30 (83.3%)
CRO	0	0	36 (100%)
COL	0	3 (8.3%)	33 (91.7%)
FEP	4 (11.1%)	2 (5.6%)	30 (83.3%)
GM	1 (2.8%)	0	35 (97.2%)
IPM	15 (41.7%)	1 (2.8%)	20 (55.6%)
LVX	17 (47.2%)	4 (11.1%)	15 (41.7%)
MPM	4 (11.1%)	0	32 (88.9%)
MI	25 (69.4%)	3 (8.3%)	8 (22.2%)
CPS	3 (8.3%)	4 (11.1%)	29 (80.6%)
SXT	6 (16.7%)	0	30 (83.3%)
TZP	1 (2.8%)	0	35 (97.2%)

## Discussion

The AST findings were analyzed for 208 participants who had positive *E*. *meningoseptica* culture reports. This retrospective study included all clinical sample types for analysis. The most effective treatment for *E*. *meningoseptica* isolates was minocycline (148, 71.2%). Other susceptible drugs were levofloxacin and imipenem. Participants with prolonged hospitalizations had fewer resistant instances. Increased mortality among those with multidrug-resistant pathogens might be the cause. Similar AST patterns were observed among *E*. *meningoseptica* isolates found in blood, ETT, and other samples. According to recent research conducted in India, E. meningoseptica infections have become increasingly prevalent in recent years [3,26].

The AST findings of this study revealed a limited number of treatment options, with some variations in the susceptibility of various antibiotics. Owing to the presence of MBLs and ESBLs, *Elizabethkingia* spp. exhibit intrinsic resistance to multiple antibiotic classes [15,16,27]. The presence of numerous chromosomally encoded MBLs sets *Elizabethkingia* spp. apart from other bacteria [27,28]. However, the lack of documented minimum inhibitory concentration breakpoints makes susceptibility testing difficult, requiring alternative techniques, such as broth microdilution, for precise determination [27-29]. Matrix-assisted laser desorption ionization-time-of-flight mass spectrometry (MALDI-TOF MS) (BioMérieux, Marcy-l'Étoile, France) should be used to determine the species of isolates that the VITEK 2 system has provisionally identified as *Elizabethkingia* [3]. Therefore, most clinical studies continue to underreport *Elizabethkingia anophelis*, which is frequently mistaken for *E*. *meningoseptica* [3]. *E*. *anophelis* got its name since it was initially found in the *Anopheles* mosquito's digestive tract [3,20]. Subsequent studies, however, demonstrated that mosquitoes were unlikely to be the transmission vector for diseases linked to *E*. *anophelis* [20,27]. Both *E*. *anophelis* and *E*. *meningoseptica* share the same reservoirs for growth in hospital settings, namely, intravenous infusions and fluids, contaminated hospital environments, and infected catheters [3,20,27].

Since there are no defined rules for reporting AST for *Elizabethkingia* in the CLSI, 2024 edition [24], the zone of inhibition for *Acinetobacter baumannii* and the prescribed antimicrobial discs were used as interpretive criteria. We found minocycline, levofloxacin, and imipenem to be effective antimicrobials against *E*. *meningoseptica* in this study. Our study findings were corroborated by several previous studies [1,2,4]. Our previous studies in the neurosurgery units also demonstrated *E*. *meningoseptica* isolates’ susceptibility towards minocycline [22,23].

Strengths and limitations

We analyzed two years of data on the AST patterns of an emerging and multidrug-resistant pathogen, i.e., *Elizabethkingia* spp. We also performed subgroup analyses by sample and length of hospitalization. The mosaic plot and chord diagrams facilitated the interpretation of the study findings. Our study also had several limitations. First, the data was collected only from the microbiology laboratory. Hence, the AST findings could not be correlated with the prescribed antimicrobials. Second, we were unable to perform MALDI-TOF MS due to its unavailability during the study period. The failure of molecular and proteomic identification of the isolates is a major limitation of this investigation. Only MALDI-TOF MS or whole-genome sequencing can correctly identify *Elizabethkingia* to the species level. Third, we had to leverage the details of *A*. *baumannii* from the CLSI to interpret the AST findings of *Elizabethkingia* spp. Fourth, the impact of comorbidities, concurrent drugs, and diagnosis on AST results could not be evaluated.

## Conclusions

The 208 blood, ETT, and other samples were found to be positive for *E*. *meningoseptica* in this retrospective study. The bacterial isolates demonstrated maximum susceptibility towards minocycline, levofloxacin, and imipenem. The highest resistance was detected against ceftriaxone, aztreonam, amikacin, and piperacillin-tazobactam. We recommend prospective studies to determine the AST pattern and horizontal transfer of resistance genes of *Elizabethkingia* spp.
